# Diversification and conservation of the extraembryonic tissues in mediating nutrient uptake during amniote development

**DOI:** 10.1111/j.1749-6632.2012.06726.x

**Published:** 2012-10-10

**Authors:** Guojun Sheng, Ann C Foley

**Affiliations:** 1 Lab for Early Embryogenesis, RIKEN Center for Developmental BiologyKobe, Hyogo 650-0047, Japan; 2 Greenberg Division of Cardiology, Weill Cornell Medical CollegeNew York, New York

**Keywords:** amniotes, mammals, birds, chorioallantoic placenta, yolk sac endoderm, nutrition, trophectoderm, extraembryonic tissues

## Abstract

The transfer of nutrients from the mother through the chorioallantoic placenta meets the nutritional needs of the embryo during human prenatal development. Although all amniotes start with a similar “tool kit” of extraembryonic tissues, an enormous diversity of extraembryonic tissue formation has evolved to accommodate embryological and physiological constraints unique to their developmental programs. A comparative knowledge of these extraembryonic tissues and their role in nutrient uptake during development is required to fully appreciate the adaptive changes in placental mammals. Here, we offer a comparative embryological perspective and propose that there are three conserved nutrient transfer routes among the amniotes. We highlight the importance of the yolk sac endoderm, thought to be a vestigial remnant of our amniote lineage, in mediating nutrient uptake during early human development. We also draw attention to the similarity between yolk sac endoderm-mediated and trophectoderm-mediated nutrient uptake.

## Introduction

Reptiles, birds, and mammals belong to the amniotes, a group of vertebrates that reproduce on land. This reproductive trait is associated with two conserved features in amniote development: internal fertilization and the formation of extraembryonic tissues. The former lays the foundation for a wide spectrum of strategies used by the amniotes to supply nutrients to their developing embryos, while the latter provides the developing embryo with an aqueous environment, access to nutrition, oxygen supply, and metabolic waste storage. The eutherian mammals represent one end of this spectrum in which the oocyte is almost devoid of yolk and the vast majority of maternal nutrients pass through a chorioallantoic placenta. On the other end of this spectrum are the birds, which have among the largest megalecithal eggs in the animal kingdom. Despite this seeming diversity, all extant amniote species constitute a narrow phylogenetic clade and their developmental processes, including those mediating nutritional uptake during embryogenesis, are remarkably conserved.

## The basic “tool kit” of extraembryonic tissues in the amniotes

**Figure 1:**
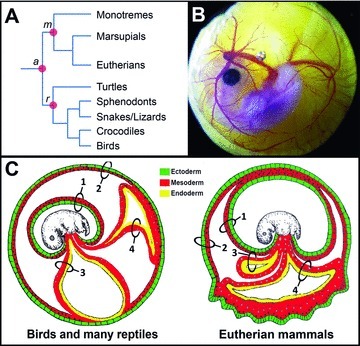
Extraembryonic tissues during amniote development. (A) A simplified phylogenetic tree of the extant amniotes. a, amniotes; m, mammals; r, reptiles (including birds). (B) A developing turtle (*Pelodiscus sinensis*) embryo, showing the embryo proper together with all associated extraembryonic tissues. (C) Two examples of how an amniote embryo organizes its extraembryonic tissues. Modified after Ferner and Mess,^3^ with permission from Elsevier. Left: schematic view of the organization in birds and many reptiles. Right: schematic view of the organization in eutherian mammals. 1, amnion; 2, chorion; 3, yolk sac; 4, allantois. Germ layers are color-coded (green, ectoderm; red, mesoderm; yellow, endoderm).

The amniotes evolved about 370 million years ago from the amphibians.^1^ Extant amniotes can be divided into two major groups: mammalia and reptilia (Fig. 1A). The group mammalia includes the prototherians (monotremes), therians (marsupials), and eutherians (true mammals); while the group reptilia includes all living reptiles (turtles, tuatara, snakes, lizards, and crocodiles) and birds. Ancestral mammals are believed to be more reptile like, and the monotremes (the basal extant mammals) share many developmental features with reptiles.^2^–^4^

A major part of amniote embryogenesis, especially in its early phase, is dedicated to the elaboration of extraembryonic tissues that are essential for survival of the developing embryo (Fig. 1B). All amniotes contain the following four extraembryonic components: the amnion, chorion, yolk sac, and allantois (Fig. 1C). Like the intraembryonic tissues, these extraembryonic tissues are composed of cells representing the three germ layers: ectoderm, mesoderm, and endoderm. The amnion is a double-layered membrane composed of inner ectoderm (facing the embryo) and outer mesoderm layers (Fig. 1C, structure 1). The amnion encircles the fluid-filled amniotic cavity that ensures an aqueous environment for embryonic development and gives the amniote its name. The chorion is also a bilayer of ectoderm and mesoderm, but with an inverted topographic arrangement compared to the amnion (Fig. 1C, structure 2). The chorion marks the external boundary of the embryo. The space between the amnion and the chorion is the extraembryonic coelomic (body) cavity. The yolk sac is comprised of both endoderm and mesoderm layers (Fig. 1C, structure 3). The yolk sac endoderm is a mono-layered epithelium and is the principal interface for nutrient uptake in megalicithal embryos. The yolk sac mesoderm is multilayered and is the major niche for hematopoietic development. The allantois is also composed of the endoderm and mesoderm (Fig. 1C, structure 4). The mesoderm of the amnion and chorion is referred to as extraembryonic somatic mesoderm because it is associated with the ectoderm and is avascular. The mesoderm of the yolk sac and allantois is called the extraembryonic splanchnic mesoderm because it is associated with the endoderm and is heavily vascularized.

## Variations in nutritional supply among the amniotes and adaptive changes in the organization of the extraembryonic tissues

These four basic extraembryonic components are extensively modified among amniote species to accommodate different developmental and environmental constraints during their embryonic growth. This diversity in developmental adaptation suggests that the amniotes are extremely flexible in modulating their extraembryonic tissues as needed. Indeed the topographic organization of the four main extraembryonic tissues is not well-conserved, even between species with close phylogenetic relationships.^5^ What remains conserved, however, can be understood from a comparative embryological perspective with regard to two major biological processes. First the organization of these tissues is heavily dependent on the source of maternal nutrient supply and the entry points of these nutrients to the developing embryo. The other major constraint on the development of extraembryonic tissues is the need to either sequester or eliminate the toxic end products of metabolism.

With this in mind, two major morphotypes of extraembryonic tissue organization can be recognized (Fig. 1C). In oviparous birds and reptiles, the yolk sac is the major site of nutrient absorption and the chorioallantoic membrane serves both as reservoir for uric acid (an end product of protein metabolism) and is responsible for gas exchange and calcium intake. As a consequence, the yolk sac is very large in these animals as it must contain enough nutrients to meet all of the needs of the embryo for the entire gestational period. Conversely, the allantois often starts off small but expands rapidly during development to meet the storage needs of the embryo and to take on its second function as a site for gas exchange. By contrast, in viviparous eutherian mammals, whose major source of nutrition is through placental attachment to the maternal blood supply, the size of the yolk sac has become greatly reduced and the chorioallantoic membrane facilitates almost all aspects of the maternofetal exchange. In between these two extremes, however, are ovoviviparous snakes, viviparous squamates with yolk sac or allantoic placentae, oviparous monotremes, and viviparous marsupials with yolk sac placentae.^3^ Even among the eutherian mammals, it has been well documented that there exists a wide spectrum of strategies used to mediate maternofetal interactions during implantation and placentation.^6^–^8^

**Figure 2:**
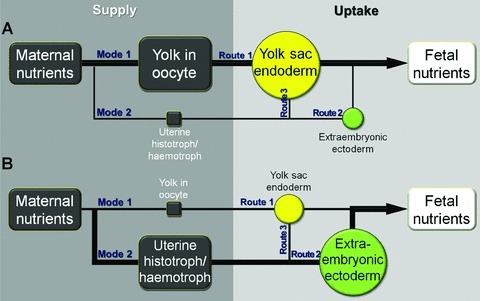
Variations in maternal nutrient supply and embryonic nutrient uptake. On the maternal side, nutrients are either deposited in the oocyte internally during its maturation (vitellogenesis) (mode 1) or provided to the developing embryo externally from the oviduct/uterus through either simple apposition of fetal and maternal tissues or complex placentation (collectively termed uterine histotroph/hemotroph here) (mode 2). Nutrients in the oocyte are taken up by the embryo through the yolk sac endoderm (route 1), and nutrients in the uterine histotroph/hemotroph are taken up by either the extraembryonic ectoderm (chorionic ectoderm or trophectoderm) (route 2) or the yolk sac endoderm (route 3). (A) In avian embryos, mode 1 and route 1 are dominant. (B) In eutherian embryos, mode 2 and route 2 are dominant. But in all amniote embryos, both maternal supply modes and all three nutrient uptake routes are operational, and the importance of route 3 transfer in mammalian embryos has been underappreciated.

As a consequence of these different tissue arrangements, the route of nutritional uptake also varies among the amniotes (Fig. 2). In egg-laying organisms, nutrients are mainly deposited in the oocyte during its maturation (mode 1; the yolk mode). In eutherian embryos, nutrients are provided to the embryo from the oviductal/uterine environment (mode 2; the uterine mode). But in practice, these two modes operate in concert in all amniotes, and this is true even in the two extreme cases mentioned above. For example, in birds, in addition to the yolk mode, maternal nutrients are supplied from the uterine environment as albumen and shell materials (Fig. 2A). In eutherian mammals, in addition to the uterine mode, the oocyte is still much larger than any somatic cell and nutrients deposited in the oocyte are vital for early cleavages after fertilization (Fig. 2B).

More importantly, for these maternal nutrients to nourish the embryo there are only two conserved entry points: the yolk sac endoderm and the extraembryonic ectoderm (Fig. 2, yellow and green, respectively). Nutrients deposited in the yolk directly face the yolk sac endoderm. They enter the embryo through the yolk nutrients → extraembryonic endoderm → extraembryonic vasculature → embryo route (Fig. 2, route 1).^9^ In the uterine environment, nutrients enter the embryo through either the extraembryonic ectoderm (uterine nutrients → extraembryonic ectoderm → extraembryonic vasculature → embryo; route 2) or the yolk sac endoderm (uterine nutrients →→ extraembryonic endoderm → extraembryonic vasculature → embryo; route 3). The chorioallantoic placenta in eutherian mammals is a typical example of route 2 transfer.^7^, ^10^, ^11^ Maternal uterine nutrients are transported to the extraembryonic vasculature through the trophectoderm via epitheliochorial, endotheliochorial, or hemochorial contact.^8^, ^12^ The route 3 transfer, although not sufficiently appreciated, also plays an important role. In eutherian mammals, it is an essential route to nourish embryonic growth before the chorioallantoic placenta becomes functional.^13^–^16^ In birds, the majority of the albumen materials are taken up through this route.^17^ In amniote groups with prominent yolk sac placenta as mentioned above, the yolk sac endoderm is a significant port of entry for uterine-derived nutrients.^3^, ^14^, ^18^, ^19^

## Nutrient transfer through the yolk sac endoderm and extraembryonic ectoderm

**Figure 3:**
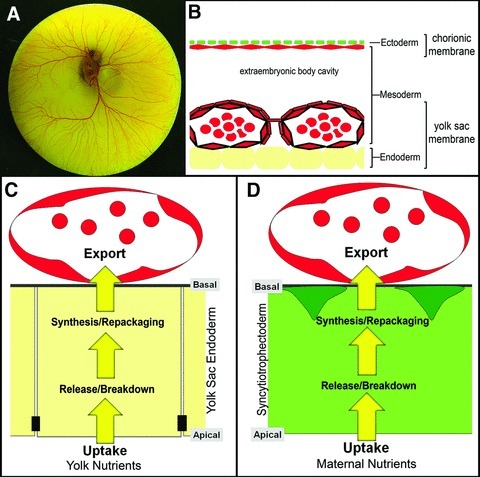
Maternal-to-fetal nutrient transfer is an active and complex process. (A) A developing chicken embryo is overshadowed by the massive yolk sac and its associated yolk sac vasculature. (B) A schematic view of the germ-layer and cell-layer organization in the extraembryonic region of a developing chicken embryo. Ectoderm, green; mesoderm, red; endoderm, yellow. Yolk, located below the endoderm, is omitted. (C) The yolk sac endoderm is an epithelium with its apical side facing the yolk and its basal side facing the extraembryonic vasculature (smooth muscle cells omitted). The endoderm cells break down and modify lipids taken up from the yolk, break down yolk proteins, and synthesize *de novo* all major serum proteins and minor regulatory molecules to be released into circulation. (D) A schematic view of the extraembryonic ectoderm- (syncytiotrophectoderm-) mediated nutrient uptake in humans in comparison with the yolk sac endoderm-mediated nutrient uptake shown in C. Light green, syncytiotrophectoderm; dark green, cytotrophectoderm cells. Despite differences in germ-layer origin and nutrient composition, there are remarkable cellular and biochemical similarities between these two processes.

In birds, the yolk sac is the predominant structure for nutrient supply (Fig. 3A). Maternal nutrients are taken up, broken down, repackaged, secreted, and transported through extraembryonic circulation to be used for embryonic growth (Figs. 3B and 3C).^9^ Yolk materials are initially enclosed by the yolk cell membrane. But soon the yolk cell membrane degenerates and yolk materials are in direct contact with the apical surface of the yolk sac endoderm.^9^, ^20^ The basal (vascular) side of the yolk sac endoderm is tightly associated with the splanchnic mesoderm that contains the vascular endothelial cells, blood cells within the vasculature and vascular smooth muscle cells around the vasculature (Fig. 3B).^9^, ^21^–^23^ The chicken yolk sac endoderm expresses high levels of genes encoding for enzymes involved in the release/breakdown and synthesis/repackaging of lipids, proteins, and carbohydrates, and for major serum proteins such as prealbumen, alpha2-macroglobulin, transferrin, and apolipoproteins as well as for hormones involved in the regulation of embryonic growth, suggesting that there is extensive lipid modification and *de novo* protein synthesis in the yolk sac endoderm (Fig. 3C).^9^ In support of this notion, it has been observed that the lipid and protein compositions of avian egg yolk are very different from those in the embryonic circulation.^24^ Together these data suggest that nutrient transfer through the yolk sac endoderm is a complex and active process and that this process is likely regulated by both extraembryonic and intraembryonic derived signals.

In eutherian mammals, the chorioallantoic membrane serves as the basic tissue for nutrient transfer during embryonic development. This structure represents a merging of the highly vascularized mesendoderm (splanchnopleure) of the allantois and the avascular chorionic somatopleure. The somatopleure consists of the extraembryonic ectoderm and a thin layer of mesothelial (smooth muscle-like) cells, the somatic extraembryonic mesoderm (Fig. 3B). Initially during development, a large extraembryonic body cavity separates this from the splanchnopleure (endoderm and splanchnic mesoderm). However, as the allantois expands it obliterates this space and the outer portion of the allantois comes into apposition to the extraembryonic somatopleure and they fuse to form the chorioallantoic membrane.

Eutherian mammals also possess a yolk sac. However, its role in embryonic nutrition has largely been ignored because it was assumed to be nothing more than a vestigial remnant. Despite this, several lines of evidence suggest that the mammalian yolk sac plays an active and complex role in endoderm-mediated nutrient uptake. Structurally, the mammalian yolk sac endoderm is very similar to the avian yolk sac endoderm and is indicative of active uptake from its apical surface and high metabolic processing within its cytoplasm.^18^, ^19^,^25^–^30^ Like its avian counterpart, the mammalian yolk sac endoderm actively produces major serum proteins.^31^–^33^ Evidence from *in vitro* cultured rat embryos also support the notion that proteins metabolized in the yolk sac endoderm can contribute to protein formation in the embryo.^34^ During human development, the vitelline (yolk sac) circulation is established at the beginning of the fourth week post-fertilization, a couple of weeks before the placental capillary network is connected to the embryonic circulation and up to a month before functional nutrient transfer from maternal blood is established.^16^, ^35^–^37^ Nutritional needs during this critical window, which encompasses late patterning and early organogenesis events during human development, are therefore most likely mediated through the yolk sac endoderm.

Unlike the avian yolk sac that is filled with nutrients during maturation, the eutherian yolk sac is essentially devoid of nutrients. How therefore can maternally derived uterine nutrients find their way into the yolk sac of a eutherian mammal? Two mechanisms have been proposed. In lab rodents, a bilaminar yolk sac placenta is functional for a significant period of their development.^14^, ^18^, ^38^ The fetal part of rodent yolk sac placenta is composed of the mural trophectoderm cells, parietal yolk sac endoderm cells, and an acellular Reichert's membrane. Both the mural trophectoderm and parietal yolk sac endoderm form incomplete layers and maternal nutrients enter the yolk sac cavity by passing through the Reichert's membrane. The visceral yolk sac endoderm, with its associated extraembryonic vasculature, then mediates nutrient uptake. In humans, the extraembryonic coelomic cavity interposes the extraembryonic ectoderm and the yolk sac. Uterine nutrients enter the yolk sac by passing through the extraembryonic ectoderm and the extraembryonic coelomic cavity.^13^, ^15^, ^16^, ^26^, ^27^, ^39^, ^40^ Once inside the yolk sac cavity, uterine nutrients are taken up by the yolk sac endoderm and transported to the embryo through the yolk sac vasculature. The exact anatomical and ultrastructural nature of this second transfer mechanism, however, has not been clearly resolved.

Finally, we come back to the chorioallantoic placenta as the main nutrient transfer route in humans and other eutherian mammals (Fig. 3D). Fetal-derived syncytiotrophectoderm or cytotrophectoderm functions as the only barrier of maternofetal exchanges for the chorioallantoic placenta. The apical side of the trophectoderm faces the maternal environment, and its basal side is associated with the extraembryonic somatic mesoderm and allantois-associated splanchnic mesoderm. The major embryological difference between this transfer route (Fig. 3D) and the yolk sac endoderm-mediated transfer routes (Fig. 3C) is the use of ectoderm-derived tissues for nutrient uptake and allantois-associated vasculature for the transport of absorbed nutrients. However, it is worth noting that this transport route evolved early in the amniote lineage, and that neither the chorionic ectoderm as the entry point for nutrient absorption, nor the allantoic vasculature as the nutrient transport venue, nor the association of these two as the main nutrient absorptive organ, is unique to the eutherian mammals. Comparison of the yolk sac-mediated nutrient uptake in birds and the chorioallantoic placenta-mediated nutrient uptake in mammals is relevant, therefore, to the cell biological, biochemical, and physiological understanding of the latter. Although a lot is known about chorioallantoic placental nutrient transport,^10^, ^11^, ^41^, ^42^ it remains poorly understood in terms of how this process is regulated by fetal and maternal (extrinsic) signals and how the trophectoderm itself (intrinsic) integrates these signals to control and modify nutritional contents during their passage. The avian yolk sac endoderm, easily accessible for experimental intervention and analysis, may be a valuable animal model for such investigations.
